# Heterologous *VvDREB2c* Expression Improves Heat Tolerance in *Arabidopsis* by Inducing Photoprotective Responses

**DOI:** 10.3390/ijms24065989

**Published:** 2023-03-22

**Authors:** Qian Zha, Xiangjing Yin, Xiaojun Xi, Aili Jiang

**Affiliations:** 1 Research Institute of Forestry and Pomology, Shanghai Academy of Agricultural Sciences, Shanghai 201403, China; zhaqian@saas.sh.cn (Q.Z.); yinxiangjing@saas.sh.cn (X.Y.); 2 Shanghai Key Labs of the Protected Horticultural Technology, Shanghai Academy of Agricultural Sciences, Shanghai 201403, China

**Keywords:** *Arabidopsis*, dehydration-responsive element, grape, heat tolerance, photoprotection

## Abstract

Extreme temperatures limit grape production and sustainability. Dehydration-responsive element-binding (DREB) transcription factors affect plant responses to temperature related stresses. Therefore, we investigated the role of *VvDREB2c*, a DREB-coding gene, found in grapes (*Vitis vinifera* L.). Protein characterization revealed that VvDREB2c is localized to the nucleus and that its AP2/ERF domain contains three β-sheets and one α-helix sheet. Analysis of the *VvDREB2c* promoter region revealed the presence of light-, hormone-, and stress-related cis-acting elements. Furthermore, we observed that the heterologous expression of *VvDREB2c* in *Arabidopsis* improved growth, drought tolerance, and heat tolerance. Furthermore, it improved the leaf quantum yield of regulated energy dissipation [Y(NPQ)], elevated the activities of RuBisCO, and phosphoenolpyruvate carboxylase and reduced the quantum yield of non-regulated energy dissipation [Y(NO)] in plants exposed to high temperatures. *VvDREB2c*-overexpressing lines also specifically upregulated several photosynthesis-related genes (*CSD2*, *HSP21*, and *MYB102*). In addition, *VvDREB2c*-overexpressing lines reduced light damage and enhanced photoprotective ability by dissipating excess light energy and converting it into heat, which eventually improves tolerance to high temperature. The contents of abscisic acid, jasmonic acid, and salicylic acid and differentially expressed genes (DEGs) in the mitogen-activated protein kinase (MAPK) signaling pathway were affected by heat stress in *VvDREB2c*-overexpressing lines, which indicated that *VvDREB2c* positively regulates heat tolerance via a hormonal pathway in *Arabidopsis*. *VvDREB2c* promotes heat tolerance in *Arabidopsis* by exerting effects on photosynthesis, hormones, and growth conditions. This study may provide useful insights into the enrichment of the heat-tolerance pathways in plants.

## 1. Introduction

In order to counter abiotic stress, plants have evolved a variety of defenses, which are activated by adverse signals and the subsequent expression of defense-related genes [1]. In particular, dehydration-responsive-element-binding (DREB) transcription factors (TFs) mediate abiotic stress responses [2]. These proteins contain a conserved DNA-binding region (the AP2/EREBP domain) that specifically binds to DRE (A/GCCGAC) cis-acting elements or similar elements with a CCCGAC core and, thereby, regulate the expression of genes harboring the corresponding promoter elements [3]. For example, DREBA1 TFs are involved in the regulation of low-temperature stress response and DREBA2 TFs are involved in the regulation of salt, drought, and heat-stress responses [4,5]. The structures and functions of the DREB genes differ among plant species [6,7].

Excessively high temperatures induce premature aging of the leaves and abnormal softening of the fruits of grapes (*Vitis vinifera* L.), an economically important perennial cash crop, thereby reducing production and causing economic losses [8,9]. Quantitative trait locus (QTL) research has been applied to determine molecular markers related to heat tolerance in several crops, such as rice and wheat [10,11]. QTL analysis on berry size and disease resistance traits has been studied in grapevines [12,13]. However, there is no QTL analysis on high-temperature resistance traits. In the current study, we aimed to identify heat-tolerance genes and determine the molecular regulation pathways linked to genes that play a role in the heat adaptation process of grape leaves.

*DREB2c* reportedly regulates heat-stress responses in grapes [14]. Heat stress promotes the degradation of *VOZ1* and the subsequent production of *DREB2c*, which subsequently activates the *HSFA3* promoter, and thereby, the thermal adaptation mechanism [15]. Similarly, in *Arabidopsis*, the terminal promoter region of *AtDREB2c* mediates tissue-specific expression under heat stress [16] and interacts with *AtCYS4*, *AtHHR2*, *ROC4*, and other genes to promote heat-stress tolerance [17]. Therefore, *DREB2c* appears to enhance the adaptability of plants to high temperatures via a complex molecular regulatory network.

High temperatures also exert physiological and biochemical effects on plant leaves. For example, photosystem II (PSII) activity plays an important role in plant responses to high-temperature stress. The primary site of thermal damage is the thylakoid membrane in the photosynthetic system, with the effect on PSII being the most prominent. High-temperature treatment reportedly reduces electron transport in the leaves, which is indicative of damage to the PSII reaction center and downstream-mediated electron transport systems [18]. In addition, high temperatures inhibit PSII repair and aggravate high-temperature stress damage [19]. Studies have found that the non-photochemical quenching (NPQ) activity of PSII in grape leaves is positively correlated with high-temperature adaptability, and also that the NPQ activity of high-temperature tolerant grape varieties is superior [8,20]. NPQ of chlorophyll fluorescence is thought to be an indicator of an essential regulation and photoprotection mechanism against stress in photosynthetic organisms. Maximal photoprotection can be achieved if NPQ is regulated in such a way that PSII reaction centers stay open under given light conditions [21].

Environmental degradation and abiotic stress increasingly limit both grape production and sustainability [9], and thus investigating the role of *DREBs* may help elucidate the mechanisms underlying heat tolerance in grapes. However, although 36 DREB TFs have been identified via genomic data [22], studies that examined their role in resistance-related functions remain limited. Even though DREB TFs of several plants have been investigated, the structures and functions of DREB TFs differ among species [7]. A previous study reported that *VvDREB2c* is significantly upregulated in response to high temperatures [20]. The objective of the present study was to characterize the corresponding gene and protein as well as to investigate the role played by *VvDREB2c* in heat-stress responses via heterologous expression in transgenic *Arabidopsis*.

## 2. Results

### 2.1. Target Gene Characterization

VvDREB2c is 1101 bp long (Supplementary File S1; OM630427). VvDREB2c together with AtDREB2c and HaDREB2c may be distinguished by the presence of two exons, rather than one. In addition, cis-acting elements of the VvDREB2c promoter region may be separated into five classes (Table 1) as follows: (i) the basic elements of the TATA- and CAAT-box motifs; (ii) light-responsive elements (e.g., Box-4, G-box, GT-1-motif, and MRE); (iii) hormone (e.g., abscisic acid, auxin, and ethylene)-related response elements; (iv) stress (e.g., heat, drought, and cold)-related response elements; and (v) other response elements (e.g., metabolism regulatory element O_2_-site and hypoxia-specific action element GC-motif).

**Figure 1:**
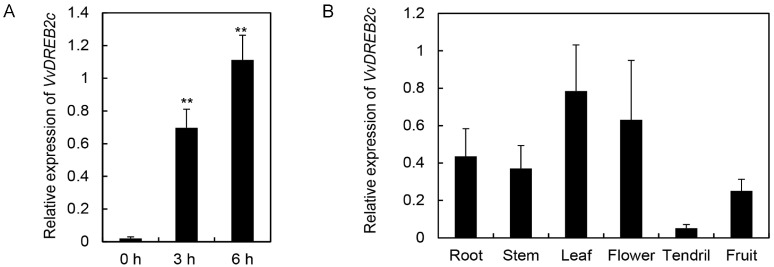
Expression of *VvDREB2c* in grapevine. (**A**) *VvDREB2c* expression in grape leaves after 3 and 6 h of high temperature (45 °C) exposure. (**B**) *VvDREB2c* expression in grapevine organs after 3 and 6 h of high temperature (45 °C) exposure. ** indicates a significant difference (*p* < 0.05).

In addition, the expression of VvDREB2c in grape leaves was significantly increased following exposure to high temperature (45 °C) for 3 and 6 h (Figure 1A) compared with that at 0 h; however, it was lower in tendrils than in other organs (Figure 1B).

### 2.2. Target Protein Characterization

**Figure 2:**
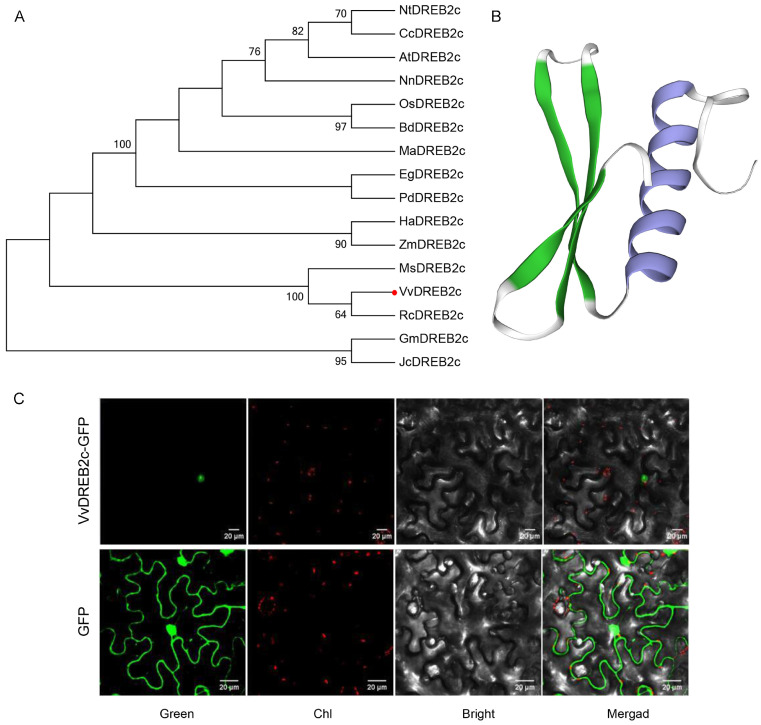
VvDREB2c sequence, structure, and localization. (**A**) Phylogenetic analysis of VvDREB2c (red dot) and DREB2c-related proteins from *Nicotiana tabacum*, *Cajanus cajan*, *Arabidopsis thaliana*, *Nelumbo nucifera*, *Oryza sativa japonica*, *Brachypodium distachyon*, *Musa acuminata*, *Elaeis guineensis*, *Phoenix dactylifera*, *Helianthus annuus*, *Zea mays*, *Malus sieversii*, *Ricinus communis*, *Glycine max*, and *Jatropha curcas*. (**B**) Prediction of VvDREB2c tertiary structure. (**C**) Subcellular localization of VvDREB2c in tobacco; Green: green fluorescent protein (GFP) signal; Chl: red signal is chlorophyll fluorescence signal; Bright: protoplast image in bright field; Merged: combination of Green and Chl images.

The predicted VvDREB2c protein was 366-amino acids (AAs) long, with a molecular weight of 39.92 kDa and an isoelectric point of 4.97. A phylogenetic analysis revealed that the amino acid sequence of VvDREB2c was highly similar to that of RcDREB2c and MsDREB2c (Figure 2A). Structural analyses revealed that VvDREB2c contained a tertiary structure (single helix structural protein subunit) at 76–132 AAs (Figure 2B), and three β-sheets and one α-helix in the AP2/ERF domain.

VvDREB2c-GFP was observed around the nucleus of tobacco cells while the control GFP was present in all tissue cells (Figure 2C), indicating that VvDREB2c was localized to the nucleus.

### 2.3. Overexpression Analysis

#### 2.3.1. VvDREB2c Expression

**Figure 3:**
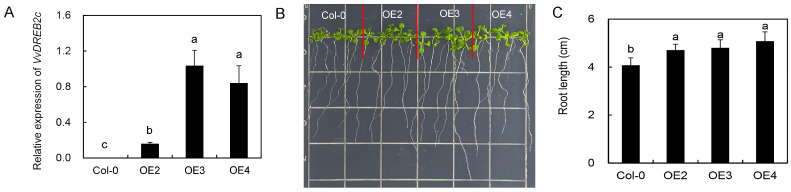
Effect of heterologous VvDREB2c expression and mannitol supplementation on VvDREB2c expression and phenotypes in Arabidopsis. (**A**) VvDREB2c expression in Col-0 and transgenic lines. (**B**) Leaves of plants grown on normal medium. (**C**) Root length of plants grown on normal medium. Different letters over bars indicate significant differences (*p* < 0.05).

The expression of VvDREB2c in Arabidopsis lines OE2, OE3, and OE4 overexpressing VvDREB2c was higher than that in Arabidopsis Columbia-0 (Col-0), indicating the complex test requirements of transgenic lines (Figure 3A). There was no trace of blue staining on Col-0 plants (Figure S2). Arabidopsis VvDREB2c -overexpressing lines OE2, OE3, and OE4 showed traces of gus staining on leaves and roots. The above results demonstrated that VvDREB2c has been successfully transformed into Arabidopsis plants.

#### 2.3.2. Plant Phenotype

The leaves of Arabidopsis grown on normal media curled following heat treatment (Figure 3B). The leaves of the transgenic lines were slightly larger than those of Col-0 plants, and the roots were significantly longer (Figure 3C), indicating that VvDREB2c overexpression may induce plant growth.

#### 2.3.3. Heat Tolerance

**Figure 4:**
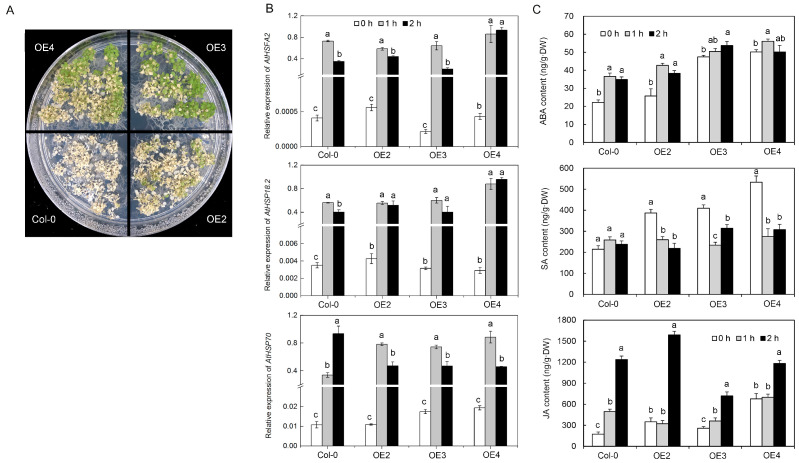
The effect of heterologous VvDREB2c expression and exposure to high temperature on phenotype, heat-stress-related gene expression, and hormone levels in Arabidopsis. (**A**) Phenotype. (**B**) AtHSFA2, AtHSP18.2 and AtHSP70 expression. (**C**) Hormone levels. Different letters over bars indicate significant difference in the same lines and under different treatments; *p* < 0.05).

The green status of the leaves of transgenic lines OE2, OE3, and OE4 was better than that of Col-0, indicating that transgenic lines were more tolerant to high temperatures than Col-0 (Figure 4A). There were no significant differences between the expression levels of AtHSFA2, AtHSP70, and AtHSP18.2 in the VvDREB2c-overexpressing lines and Col-0 plants (Figure 4B). However, the expression of these genes (AtHSFA2, AtHSP70, and AtHSP18.2) was strongly induced by high temperatures. These results indicated that although AtHSFA2, AtHSP70, and AtHSP18.2 may play important roles in high-temperature stress response, but they were not closely associated with DREB2c.

#### 2.3.4. Hormone Levels

The levels of ABA, SA, and JA were generally higher in the transgenic lines than in Col-0 plants (Figure 4C). The ABA content in the four lines increased after being subjected to high-temperature treatment, where it was more pronounced in the Col-0 and OE2 lines. Although there was no difference between the SA contents of Col-0 lines following high-temperature treatment, the SA contents in OE2, OE3, and OE4 lines were decreased. The JA content in all four lines increased after high-temperature treatment, and the JA content of the plants increased significantly after 2 h of high-temperature treatment.

#### 2.3.5. Photoprotection Response

**Figure 5:**
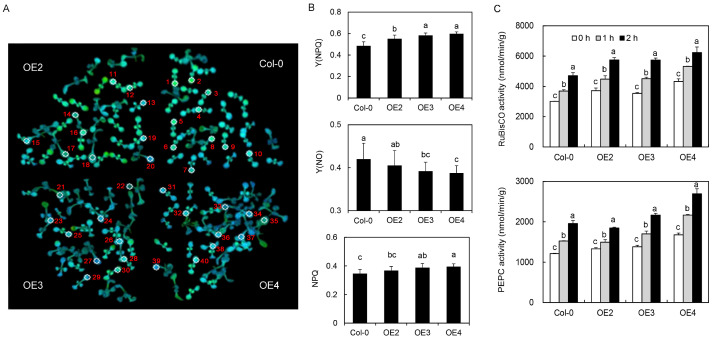
Analysis of chlorophyll a fluorescence in different lines under high-temperature treatment. (**A**) Chlorophyll a fluorescence imaging. (**B**) Changes in chlorophyll a fluorescence parameters of four lines under heat treatment. Different letters over bars indicate significant differences among different lines (*p* < 0.05). (**C**) RuBisCO and PEPC activities in the four lines under high-temperature treatment. Different letters over bars indicate significant difference in the same lines and under different treatments (*p* < 0.05).

After 2 h of high-temperature treatment, the quantum yield of regulated energy dissipation [Y(NPQ)] of transgenic lines OE2, OE3, and OE4 was significantly higher than that of Col-0, and the nonphotochemical quenching (NPQ) of transgenic lines OE3 and OE4 (Figure 5A,B). The quantum yield of non-regulated energy dissipation [Y(NO)] of transgenic lines OE3 and OE4 was significantly lower than that of Col-0. The RuBisCO and PEPC activities of transgenic lines OE2, OE3, and OE4 were higher than those of Col-0, and were significantly increased at one and two hours after high-temperature treatment (Figure 5C).

### 2.4. RNA-Seq

**Figure 6:**
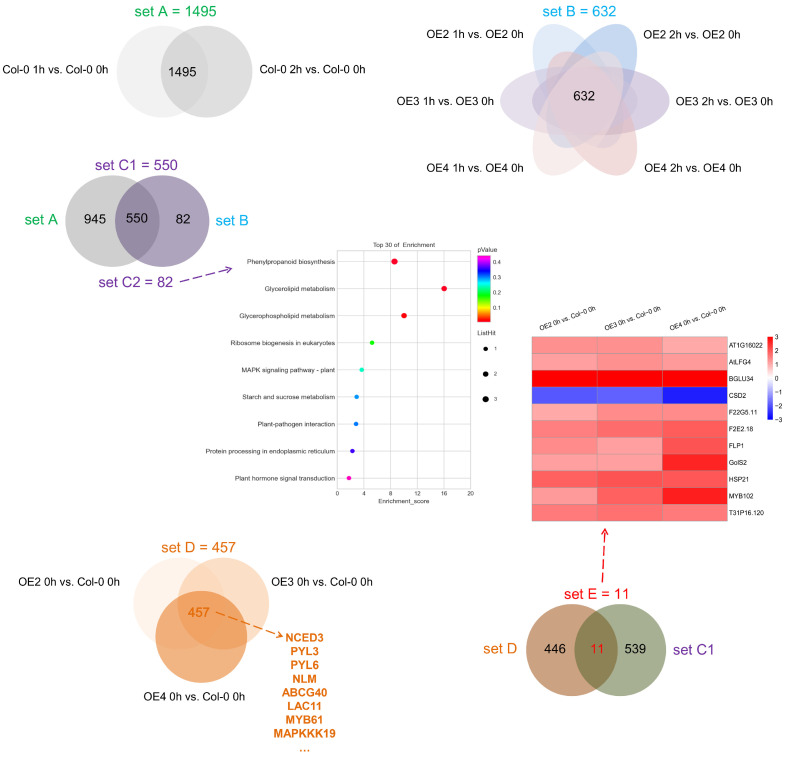
Differentially expressed genes (DEGs) identified by comparing between normal and high-temperature-exposed control (Col-0) and VvDREB2c-transgenic Arabidopsis. Set A: DEGs from normal and high-temperature-treated Col-0 (Col-0 1 h vs. Col-0 0 h and Col-0 2 h vs. Col-0 0 h); Set B: DEGs from normal and high-temperature-treated transgenic lines (OE2 1 h vs. OE2 0 h, OE2 2 h vs. OE2 0 h, OE3 1 h vs. OE3 0 h, OE3 2 h vs. OE3 0 h, OE4 1 h vs. OE4 0 h, and OE4 2 h vs. OE4 0 h); Set C1: DEGs common to sets A and B; Set C2: DEGs from set B, excluding those in set A; Set D: DEGs because of the overexpression of VvDREB2c (OE2 0 h vs. Col-0 0 h, OE3 0 h vs. Col-0 0 h, and OE4 0 h vs. Col-0 0 h). Set E: obtained using sets C and D; DEGs not only because of the overexpression of VvDREB2c, but also induced by high temperature in the four lines.

Transcriptome analysis revealed genes that were differentially expressed in Col-0 under normal and high-temperature treatments (set A), transgenic lines under normal and high-temperature treatments (set B), both Col-0 and transgenic lines under normal and high-temperature treatments (set C1), and only transgenic lines under normal and high-temperature treatment (set C2). The genes in set D comprised some differentially expressed genes (DEGs) associated with the overexpression of VvDREB2c. The genes in set E comprised not only DEGs associated with the overexpression of VvDREB2c, but also genes int the four lines that were induced by high temperature (set E; Figure 6, Table S4).

The KEGG pathway enrichment analysis indicated that set C2 (82 DEGs, the expression levels of which were induced by high-temperature exposure in the transgenic lines alone) was enriched in phenylpropanoid biosynthesis (ath00940), glycerolipid metabolism (ath00561), glycerophospholipid metabolism (ath00564), ribosome biogenesis in eukaryotes (ath03008), and the MAPK signaling pathway (ath04016; Figure 5, Table S5). In addition, the GO enrichment analysis of set C2 showed that the DEGs were enriched in biological processes and molecular functions (Figure S3), where among other functions, the DEGs had roles in mitochondrial calcium ion homeostasis, uniporter activity, and anion transport.

Many of the DEGs in set D were associated with the ABA signaling pathway (e.g., MAKPPP19, PYL3, NCED3, and LAC11), while set E comprised 11 DEGs, including one downregulated gene (CSD2) and 10 upregulated genes (AT1G16022, AtLFG4, BGLU34, F22G5.11, F2E2.18, FLP1, GolS2, HSP21, MYB102, and T31P16.120).

**Figure 7:**
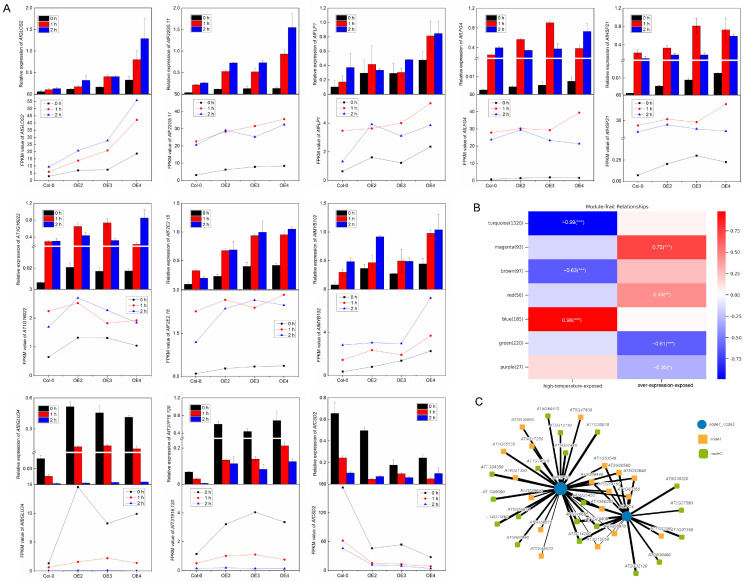
DEG analysis. (**A**): Relative expression (qRT-PCR) and FPKM value of 11 genes in different lines (Col-0, OE2, OE3, OE4) under heat stress; (**B**): Heat map of character module correlation; (**C**): Hub network of AtHSP21 and AtLFG4. The *, **, and *** indicates a significant difference (*p* < 0.05, *p* < 0.01, and *p* < 0.001, respectively).

The qRT-PCR results of 11 genes in setE are shown (Figure 7A). AtGLOS2, AtF22G5.11, AtFLP1, AtLFG4, AtHSP21, AT1G16022, AtF2E2.18, and AtMYB102 in the VvDREB2c-overexpressing lines were highly expressed under high-temperature treatment, compared to those in Col-0. AtBGLU34 and AtT31916.120 showed higher expression levels in the overexpression lines than in Col-0 and were decreased by high-temperature treatment. AtCSD2 showed lower expression levels in the overexpression lines compared to Col-0 and were decreased by high-temperature treatment. Comparison of qRT-PCR (column chart) and the FPKM values (line chart) indicated that the results of the two groups were relatively consistent, which suggested that the transcriptome data were accurate.

Cluster analysis of the DEGs was performed via Weighted Gene Co-Expression Network Analysis (WGCNA). The original data included a total of 27,444 genes and 36 samples. Genes with low fluctuations in expression (standard deviation ≤ 0.5) were filtered, leaving 2108 genes and 36 samples. A cluster heat map of 2108 genes is shown (Figure S4). Based on the selected power value, a weighted co-expression network model was established; the 2108 genes were ultimately divided into eight modules (Table S6). The gray module was represented by a gene set of that could not be attributed to any module and had no reference significance. The correlation heat map of the trait modules for the remaining seven modules (Figure 7B) indicated that the correlation of turquoise and blue modules were high. Therefore, we conducted intersection gene analysis of these two modules (Table S7) and set E to obtain two key genes, AtLFG4 and AtHSP21. A Hub network analysis of AtLFG4 and AtHSP21 was performed (Figure 7C). Most of the connecting genes were HSP proteins (Table S8), which may act as a complex in response to plant adaptation to high temperature.

## 3. Discussion

### 3.1. Gene and Protein Characterization

*VvDREB2c* is 1101 bp long and harbors two exons and its corresponding protein is 366 AAs long, with an AP2 conserved domain, indicating that this gene belongs to the AP2 gene family and is homologous to *AtDREB2c* in *Arabidopsis* [23]. VvDREB2c was also localized to the nucleus. The short-conserved sequences (PAKGSKKG, red in Supplementary File S1) directly flanking the AP2/ERF domain of DREB may function as a nuclear localization signal (NLS) [3,24].

### 3.2. The Role of VvDREB2c in Plant Heat Tolerance

Recent studies have described that DREBs are key regulators of abiotic and biotic stress responses in plants [25,26,27,28,29,30,31]. Indeed, the present study confirmed that *VvDREB2c* was closely associated with high-temperature exposure, which observation was substantiated by the findings pertaining to *AtDREB2c* in *Arabidopsis* [23] and *MsDREB2c* in *Malus sieversii* Roem [32]. In addition, the present study demonstrated that *VvDREB2c* was rapidly upregulated in response to high-temperature exposure, indicating that this gene may act as a positive regulator of the heat-stress response (Figure 5). Its overexpression improved high-temperature stress tolerance (Figure 4) in a manner-similar to that seen in *AtDREB2c* overexpression [15], and upregulated the high-temperature stress-related genes, *AtHSFA2*, *AtHSP70*, and *AtHSP18.2*.

Another important mechanism contributing to the high-temperature tolerance of the transgenic lines involves hormone pathways. High-temperature treatment was reported to trigger hormone pathways [33], and in the present study, *VvDREB2c* overexpression resulted in increased ABA, SA, and JA concentrations. In addition, 10 DEGs in set C2 were found to be involved in the MAPK signaling pathway (ath04016; Figure 6), which is directly associated with hormone regulation.

Furthermore, plant and root growth were also enhanced by *VvDREB2c* overexpression (Figure 3), possibly due to the increased heat tolerance. Transgenic plants were healthier and had longer roots, suggesting that transgenic lines may tolerate heat stress, which finding was consistent with that of Wijewardene et al. [34]. Interestingly, *BGLU34*, which was specifically and highly expressed in the transgenic lines (Figure 3), encodes β-glycosidases that contribute to glucosinolate degradation and mediate the regulation of root growth [35].

*AtLFG4* and *AtGLOS2* were also specifically and highly expressed in the transgenic lines (Figure 6). *AtLFG4* (lifeguard), which belongs to a cytoprotective family, regulates apoptosis [36], while *GLOS2* (galactinol synthase) plays a key role in the biosynthesis of raffinose oligosaccharides and soluble carbohydrates [37]. The accumulation of carbohydrates in plants is associated with tolerance to environmental stresses, such as heat and dehydration [38], which indicates that processes such as apoptosis and carbohydrate accumulation are also involved in plant heat tolerance. WGCNA analysis indicated that *AtFLG4*, *AtHSP21*, and multiple HSPs may form a protein complex that plays a role in the high-temperature adaptability of *VvDREB2c*-overexpressing lines. Kim et al. [39] found that the increased expression of HSPs was correlated with acquired thermotolerance and increased cytoprotection. Thus, the pathways suspected of involvement in these processes may warrant further study.

### 3.3. Heat Tolerance and Photoprotective Ability

The Y(NPQ) of the transgenic lines was higher than that of the control line (Figure 5B), which indicated that the transgenic lines were more tolerant of excessive light intensity and could protect themselves through self-regulation. A higher Y(NO) indicates the regulation of photochemical energy conversion and insufficient protection. Plant leaves cannot consume absorbed light energy [40]. NPQ reflects the ability of plants to dissipate excess light energy into heat, thereby indicating their light protective capacity [41]. The Y(NO) of the transgenic lines was lower, whereas the NPQ of the transgenic lines was higher than those of the control line. This indicated that *VvDREB2c* was capable of enhancing plant heat tolerance and possessed enhanced photoprotective ability.

RuBisCO is a dual-function enzyme that regulates photosynthesis and photorespiration, thereby determining net photosynthesis [42], whereas PEPC activity helps concentrate CO_2_ in C4 plants, thereby improving photosynthetic efficiency [43]. The relatively greater RuBisCO and PEPC activities in the transgenic lines indicated that the transgenic lines had a stronger ability to assimilate carbon and a stronger photosynthetic capacity than the control line. The increase in RuBisCO and PEPC activities under high-temperature treatment indicates that high temperature induces plants to respond to stressors via photoprotection and that photoprotective ability plays an important role in heat tolerance.

Among the 11 genes specifically and differentially expressed in these *VvDREB2c*-overexpressing lines (Figure 6; heatmap), the genes associated with photosynthesis were *CSD2* (chloroplastic copper/zinc superoxide dismutase), which encodes a chloroplastic Cu/Zn-SOD and increases tolerance to photooxidative stress in *Arabidopsis* [44], *HSP21*, which is closely related to the PSII activity in plant leaf photosynthesis [45], and *MYB102*, which, in rice, delays chloroplast apoptosis by downregulating the ABA accumulation signal induction pathway, thereby delaying leaf senescence and prolonging photosynthesis [46]. Thus, *VvDREB2c* may regulate photosynthesis by influencing these genes (*CSD2*, *HSP21*, and *MYB102*), thereby enhancing the heat tolerance of *VvDREB2c*-overexpressing lines. The cis-acting elements of the *VvDREB2c* promoter region also include many photosynthesis-related elements, such as the Box-4, G-Box, GT-1, and MRE motifs (Table 1). These results demonstrate that photoprotective ability is closely associated with heat tolerance in *VvDREB2c*-overexpressing *Arabidopsis* lines. Considered together, the findings of this study provide additional information that may enable the regulatory pathways of plant leaf heat tolerance to be analyzed in depth. However, photosynthesis involves many signaling and protein pathways, and therefore an in-depth analysis of the proteins and signaling linked to the photosynthetic pathway associated with *VvDREB2c* may be warranted.

## 4. Materials and Methods

### 4.1. Target Gene Sequencing and Analysis

The full-length open reading frames (ORFs) of *VvDREB2c* were cloned using transcript BLAST in *V. vinifera* (https://blast.ncbi.nlm.nih.gov/Blast.cgi, accessed on 1 May 2020). Full-length primers (VvDREB2c-F1: 5′-ATGGATACCTGCGTTCAAG-3′; VvDREB2c-R1: 5′-GAACCCCATATCTGATA-3′) were designed based on the predicted genome sequence of *DREB2c*, using cDNA from Kyoho grape as a template. Furthermore, the sequences were compared using DNAMAN (Lynnon Biosoft, San Ramon, CA, USA) and submitted to the National Center for Biotechnology Information (http://www.ncbi.nlm.nih.gov/, accessed on 17 October 2022) to obtain a GenBank accession number (BankIt2544037 *VvDREB2c* OM630427). The promoter analysis was conducted using PlantCARE (http://bioinformatics.psb.ugent.be/webtools/plantcare/html/, accessed on 1 May 2020) [47].

### 4.2. Protein Characterization

#### 4.2.1. Phylogenetic Analysis

For purposes of phylogenetic analysis, DREB2c-related protein sequences from 15 species were downloaded from NCBI (http://www.ncbi.nlm.nih.gov/, accessed on 1 May 2020): AtDREB2c (NP_565929.1), EgDREB2c (XP_010940672.1), GmDREB2c (NP_001240942), HaDREB2c (XP_021977297.1), MaDREB2c (XP_009391184.1), NtDREB2c (XP_015651102.1), OsDREB2c (XP_015651102.1), PdDREB2c (XP_008796422.1), ZmDREB2c (XP_008664551.1), MsDREB2c (JQ790526), BdDREB2c (XP_003571275.1), CcDREB2c (KYP44306.1), JcDREB2c (XP_012086678.1), NnDREB2c (XP_010243654.1), and RcDREB2c (XP_002520794.1). The sequences were then aligned using ClustalW and analyzed using MEGA version 7.0 [48].

#### 4.2.2. Bioinformatic Analysis

Tertiary structure modeling of the VvDREB2c proteins was performed using SWISS-MODEL (https://swissmodel.expasy.org/interactive, accessed on 1 May 2020).

#### 4.2.3. Subcellular Localization

A subcellular localization expression vector (pCAMBIA1301-osgfp-ky::VvDREB2c::GFP) was constructed using the pCAMBIA1301-osgfp-ky vector (Figure S1A), primers XbaDREB2c-F1 (5′-GCTCTAGAATGGATACCTGCGTTCAAG-3′) and BamDREB2c-R1 (5′-CGGGATCCGAACCCCATATCTGATA -3′), and the enzymes *XbaI* and *BamHI*. The constructed vector plasmid was then transferred into *Agrobacterium GV3101*. Finally, *Agrobacterium* suspension was injected into tobacco seedlings (*Nicotiana rustica* var Pavonii), following which the plants were cultured under low-light conditions for 2 days, after which the injected tobacco leaves were observed and photographed using a confocal microscope (C2-ER; Nikon, Minato City, Japan). The excitation and emission wavelengths of the chloroplast fluorescence signal were 640 and 675 nm, respectively, whereas those of the GFP fluorescent proteins were 488 and 510 nm.

### 4.3. Plant Materials and Growing Conditions

#### 4.3.1. Grapevine

A 1-year-old grapevine (*Vitis vinifera* L. Kyoho) that had been grown in a 1:1 potting mixture of coconut bran and humus was used as test material. Grapevines at a similar maturity stage (20–25 functional leaves) were placed in an intelligent artificial climate incubator (Qianjiang Instrument Equipment Co., Ltd., Hangzhou, China) and grown under a 14 h photoperiod (200 μmol·m^–2^·s^−1^), with light and dark temperatures of 25 ± 1 °C and 20 ± 1 °C, respectively, and 65–70% relative humidity; they were watered every other day.

#### 4.3.2. Wild-Type *Arabidopsis*

Sterilized *Arabidopsis Columbia-0* (*Col-0*) seeds were evenly planted on Murashige and Skoog (pH 5.8) plates, transferred to an artificial climate chamber, and then maintained under a 14 h photoperiod (100 μmol·m^–2^·s^−1^), with light and dark temperatures of 25 ± 1 °C and 20 ± 1 °C, respectively, and 65–70% relative humidity.

#### 4.3.3. Transgenic *Arabidopsis*

A pCAMBIA2301-ky::VvDREB2c vector was constructed using a pCAMBIA2301 vector (Figure S1B), primers XbaDRE2c-F1 and BamDREB2c-R1, and enzymes *XbaI* and *BamHI*. *Agrobacterium*-mediated *Arabidopsis thaliana* transformation was conducted using the floral dip method [49], and positive seedlings were screened and verified using polymerase chain reaction (PCR) and 30 μg/mL hygromycin 1/2 Mrashige and Skoog resistance plates. Sterilized *Arabidopsis* T3 seeds were evenly planted on Murashige and Skoog plates (pH 5.8).

##### 4.3.4. β-Glucuronidase (GUS) Histochemical Staining

The *Arabidopsis* plant was immersed in the GUS dye containing X-Gluc (50×) and GUS stain buffer and incubated at 37 °C for 24 h. Following decolorization via 70% alcohol, tissues were observed using a Leica microscope (Leica, Teaneck, NJ, USA).

### 4.4. High-Temperature Treatment

Grapevines, with six plants in each treatment group replicated thrice, were treated via exposure to 25 and 45 °C, and 7–9 functional leaves of the new shoot were collected at 0, 3, and 6 h, transferred into liquid nitrogen, and stored at −80 °C for subsequent analysis.

Concurrently, 7-day-old *Col-0* seedlings were placed at 42 °C for 2 h, and samples collected at 0, 1, and 2 h were stored and analyzed [50]. The plants were subsequently returned to normal culture conditions and observed for an additional 5 days.

### 4.5. Expression Analysis

#### 4.5.1. RNA Extraction and cDNA Generation

Total RNA from the leaves was extracted using an Omega Plant RNA Kit (Bio-Tek, Doraville, GA, USA), and the resulting purified RNA was quantified via 1% agarose electrophoresis and a UV spectrophotometer (NANO DROP2000; Thermo Scientific, San Diego, CA, USA). First-strand cDNA was then generated using 1 μg of RNA and a PrimeScript RT reagent Kit with gDNA Erase (Perfect Real Time; TaKaRa, Tokyo, Japan).

#### 4.5.2. Quantitative Real-Time PCR

Real-time PCR was performed using Roche LightCycler 480II, with 20 μL reaction mixtures, containing 10 μL of SYBR Premix Ex TaqTM (2×), 1 μL of cDNA, 0.3 μL of upstream and downstream primers (10 μmoL/L; Table S1), and 8.4 μL of ddH_2_O, with three replicates per sample. The relative expression levels of the target genes (*AtHSFA2*, *AtHSP18.2*, *AtHSP70*, and *VvDREB2c*) were calculated using geNorm [51], based on a method derived from algorithms outlined using *VvEF1*γand *VvGAPDH* as the reference genes in grape [52] and *AtUBQ10* and *AtACTIN2* as the reference genes in *Arabidopsis* [53]. The primers of *AtHSFA2*, *AtHSP18.2*, *AtHSP70*, and *AtHSP21* were from Ikeda et al. [54]. The primers of other genes that were designed and met the following conditions: the slope of standard curve was maintained at approximately −3.4 and the efficiencies of amplification between >90% and <110%.

### 4.6. Root Length Test

*Col-0* and *VvDREB2c*-transgenic *Arabidopsis* plants were grown in 1/2 Murashige and Skoog medium for 7 days and then in Murashige and Skoog medium. Plant growth was photographed, and root length was measured after 7 days.

### 4.7. Hormone Extraction and Quantification

Hormones were extracted from leaf samples using chloroform and sonication [55]. The mass spectroscopy (MS) system comprised a Waters Xevo TQ-S MS system (Waters Co., Milford, MA, USA), an electrospray ionization (ESI) source, and a MassLynx 4.1 software workstation. The chromatographic system consisted of a Waters ultra-high performance liquid chromatograph with a CORTECS UPLC T3 liquid chromatography column (2.1 mm × 100 mm, 1.6 μm). The column temperature was 40 °C and the injection volume was 2 μL. Gradient elution was achieved using mobile phases A (0.1% formic acid: water solution) and B (0.1% formic acid: methanol solution) as follows: 0 min A/B (95:5, *v*/*v*), 3 min A/B (30:70, *v*/*v*), 6 min A/B (30:70, *v*/*v*), 6.01 min A/B (95:5, *v*/*v*), and 8 min A/B (95:5, *v*/*v*).

Quantitative detection of abscisic acid (ABA), jasmonic acid (JA), and salicylic acid (SA) in the leaves was conducted via ultra-high performance liquid chromatography-electrospray ionization-tandem mass spectrometry (UPLC-ESI-MS/MS). The MS system included an API4500 triple quadrupole MS/MS detection system (AB Sciex Co., Framingham, MA, USA). All hormones were quantified via comparison with standards (Table S2) using standard curve conversion and linear calculation (Table S3).

### 4.8. Photosynthesis Analysis

#### 4.8.1. Chlorophyll a Quantification

The *Arabidopsis* lines plate was shaded and covered after heat treatment (42 °C) for 100 min, following which the chlorophyll *a* fluorescence content in the plates was measured after heat treatment (42 °C) for 2 h. An *M*-series modulation chlorophyll *a* fluorescence imaging system (IMAGING-PAM; Zealquest Scientific Technology Co., Ltd., Shanghai, China) was used to measure chlorophyll *a* fluorescence [56]. The images were exported and observed using ImagingWinGigE software (System’s own software V2.45i), and 10 data points from each treatment were selected for further analysis.

#### 4.8.2. RuBisCO and Phosphoenolpyruvate Carboxylase (PEPC) Activity Assay

RuBisCO activity (EC 4.1.1.39) was quantified using a YX-W-C703 Kit (Sino Best Biological Technology Co., Ltd., Shanghai, China), according to the method described by Zha et al., [8]. NADH oxidation rate, which ultimately reflects RuBisCO activity, was quantified at 340 nm. Meanwhile, PEPC activity (EC 4.1.4.31) was quantified using the YX-W-B203 Kit (Sino Best Biological Technology, Beijing, China). PEPC activity was assayed spectrophotometrically at 340 nm by calculating the rate of NADH decrease.

#### 4.8.3. RNA-Sequencing (RNA-Seq) Analysis

For RNA-Seq of *Col-0* and transgenic *Arabidopsis*, total RNA from the leaves was extracted using a mirVana miRNA Isolation Kit (Ambion-1561; Ambion, Inc., Austin, TX, USA), following the manufacturer’s protocol, and RNA integrity was evaluated using an Agilent 2100 Bioanalyzer (Agilent Technologies, Santa Clara, CA, USA). DNA libraries were then constructed using samples with an RNA integrity number (RIN) of ≥7 and a TruSeq Stranded mRNA LT Sample Prep Kit (Illumina, Inc., San Diego, CA, USA), and sequenced using an Illumina HiSeq 4000 sequencing platform.

Transcriptome sequencing and corresponding bioinformatic analysis were conducted by OE Biotech Co., Ltd. (Shanghai, China). Raw data (raw reads) were processed and mapped to the *Arabidopsis* reference genome (ftp://ftp.ncbi.nih.gov/genomes/Arabidopsis_thaliana/, accessed on 29 May 2021) using hisat2 [57]. The processed dataset has been deposited in the National Center for Biotechnology Information Sequence Read Archive (http://www.ncbi.nlm.nih.gov/sra/, accessed on 29 October 2021). no. PRJNA776092).

For transcript-level quantification, fragments per kilobase per million (FPKM) and read count values were calculated for each transcript using bowtie2 [58] and eXpress [59]. Differentially expressed genes (DEGs) were identified via “estimate SizeFactors” and “nbinom Test” functions in DESeq [60], using significance and differential expression thresholds of *p* < 0.05 and >2- or <0.50-fold, respectively. Hierarchical cluster analysis of the DEGs was performed to investigate transcript expression patterns, and both Gene Ontology (GO) term and Kyoto Encyclopedia of Genes and Genomes (KEGG) pathway enrichment analyses were performed using R, based on a hypergeometric distribution [61]. Venn diagrams were used to visually represent the overlap of element sets. Heatmaps were plotted by taking the logarithm of FPKM to the base 2. Bioinformatics analysis was performed using OECloud tools (https://cloud.oebiotech.cn, accessed on 29 October 2021). WGCNA was performed using OECloud tools (https://cloud.oebiotech.com/task/detail/wgcna-oehw/, accessed on 15 March 2023), and overexpression and high-temperature treatments were selected as two key traits for correlation analysis.

### 4.9. Statistical Analysis

The data were expressed as the mean ± standard deviation (SD) and analyzed using a one-way analysis of variance (ANOVA) in SPSS 15.0 (SPSS Inc., Chicago, IL, USA); statistical significance was set as *p* < 0.05.

## 5. Conclusions

The results of this study provide genetic evidence that indicates that *VvDREB2c* may play a positive role in the regulation of heat tolerance in plants by mediating photoprotective ability, hormone pathways, and growth conditions. It shows that the stable operation of leaf photosynthetic performance is an important response initiated by plants to resist adversity. Grapes showing stable photosynthetic capacity should be selected as breeding material that can be used to generate new heat-tolerant grape varieties.

## Figures and Tables

**Table 1:** Distribution of promoter elements in the promoter region of VvDREB2c in grape.

Component Function	Cis-Acting Element	Number
Basic action element	TATA-box	22
	CAAT-box	24
Photoresponse element	Box-4	3
	G-box	6
	GT-1-motif	1
	MRE	1
ABA response element	AAGAA-motif	1
	ABRE	5
Auxin response element	As-1-bo	1
	AuxRR-core	1
Methyl-JA response element	CGTCA-motif	1
	TGACG-motif	1
Ethylene response element	W-box	2
	ERE-box	2
GAs response element	F-box	1
	P-box	1
SA response element	TCA-element	5
Heat response element	AT-rich-sequence	1
Drought response element	MYB	5
Chilling response element	LTR	1
Stress response element	WRE3	2
	STRE	1
Damage-inducing element	WUN-motif	2
Response elements involved in defense and stress response	TC-rich repeats	1
Metabolic regulatory elements	O_2_-site	1
Hypoxia-specific action element	GC-motif	1

## Data Availability

The clean dataset was deposited in the National Center for Biotechnology Information Sequence Read Archive (http://www.ncbi.nlm.nih.gov/sra/ (accessed on 29 October 2021) under the accession number PRJNA776092.

## Supplementary Materials

The supporting information can be downloaded at: https://www.mdpi.com/article/10.3390/ijms24065989/s1.
