# Supplementary material for: Heterologous VvDREB2c Expression Improves Heat Tolerance in Arabidopsis by Inducing Photoprotective Responses
Source: Int J Mol Sci. 2023 Mar 22;24(6):5989. doi: 10.3390/ijms24065989 (PMC10053783; doi:10.3390/ijms24065989)
Supplement: Supplementary file 1 [file ijms-24-05989-s001.zip › Supplementary File S1.pdf]

Supplementary file S1. The sequence and protein of VvDREB2c.

>VvDREB2c sequence

ATGGATACCTGCGTTCAAGGTTCTAATAAGACTTCTTTTCCATTTGCTTCTTCCAGGAAG
AGAAAGTCTCGAAGCCGGCGAAGTGGACCTAATTCCGTGGCCGAGACTCTTGCAAGA
TGGAAACAATACAACGATATACTTGATTCTGTCCGCAAAGCTCCAGCAAAAGGTTCAA
AGAAGGGGTGTATGAAAGGTAAAGGGGGGCCCGAGAATTCAATATGTGGTTACAGGG
GTGTGAGGCAGAGGACATGGGGTAAATGGGTTGCTGAGATTCGGGAGCCAAACAGAG
GGAGTAGGCTATGGTTAGGTACCTTCCCAACTGCCATTGAAGCTGCTCTTGCATATGAC
GAAGCTGCAAGGGCCATGTATGGTTCTTCTGCCCGTCTTAATCTTCCAAATTACACCAC
GTCTTTGAAGGATTCTTCTTCGGCTCCAACTACATCGGTTTCTGATTCCACCACGACAA
CATCCAACTACTCTGAAGTGTGTGCATATGAGGATTCAAAGAAGCCTGTTTTACCGAGT
ATCAAACATGAAAGTGGCGAAGGTGAATCAGGAATAAGTGGTGGTATGCTTTCTGCAG
TGGTTAAAGCTGAACCTGCCACACCAGTTAGTTTGGTAACACAGGGAGGAGGTAATGA
TCCTGTCAATGTAGGTAATGAACCTGTTGATGCAATGAAGCTCCAGCATGAAGAAAAC
GGTCATTCTTTGGATGCCATGTACTTCAAGAATGAAGACGGAGGACAGGATTTCTTGG
AAGGATTTCCTATGGATGAAATGTTTGATGTGGATGAATTTTTGAGGGCCATAGACTCT
GACCCCCTTGCTAGCTATGGTACAAGGCAGGAATTGGGTCATGATTCTGGGCAAGTAG
GGAGCTTTGAAACTGATAATATGCAGTGGGAAAAGCCAACGGATTTATCTTACCAACTG
CAAAATCCAGATGCTAAGCTTCTTGGGAGTTTGAATCATATGGAGCAAGTGCCTTCTGA
CTTTGATTATTGCTATGGCTTCTTGCAGCCAGGCAAGCAGTTAGATCCCTGTATAGGGTT
GAATGATCAGGGGTTACTTGATTTGGAATTATCAGATATGGGGTTCTAA

>VvDREB2c protein

MDTCVQGSNKTSFPFASSRKRKSRSRRSGPNSVAETLARWKQYNDILDSVRKAPAKGSKK
GCMKGKGGPENSICGYRGVRQRTWGKWVAEIREPNRGSRLWLGTFPTAIEAALAYDEAA
RAMYGSSARLNLPNYTTSLKDSSSAPTTSVSDSTTTTSNYSEVCAYEDSKKPVLPSIKHES
GEGESGISGGMLSAVVKAEPATPVSLVTQGGGNDPVNVGNEPVDAMKLQHEENGHSLDA
MYFKNEDGGQDFLEGFPMDEMFDVDEFLRAIDSDPLASYGTRQELGHDSGQVGSFETDN
MQWEKPTDLSYQLQNPDAKLLGSLNHMEQVPSDFDYCYGFLQPGKQLDPCIGLNDQGL
LDLELSDMGF*
