# Supplementary material for: Heterologous VvDREB2c Expression Improves Heat Tolerance in Arabidopsis by Inducing Photoprotective Responses
Source: Int J Mol Sci. 2023 Mar 22;24(6):5989. doi: 10.3390/ijms24065989 (PMC10053783; doi:10.3390/ijms24065989)
Supplement: Supplementary file 1 [file ijms-24-05989-s001.zip › Table S1.pdf]

Table S1. Amplifying transcript of gene by quantitative PCR

Gene name	Primer sequence		Ref
	Forward	Reverse	
VvDREB2c	ATGGATACCTGCGTTCAAGGTTCT	TTAGAACCCCATATCTGATAATTCCAAA	Designed
VvGAPDH	TTCCGTGTTCCTACTGTTG	CCTCTGACTCCTCCTTGAT	Guillaumie
VvEF1γ	CAAGAGAAACCATCCCTAGCTG	TCAATCTGTCTAGGAAAGGAAG	et al., 2013
AtACTIN2	CTTGCACCAAGCAGCATGAA	CCGATCCAGACACTGTACTTCCTT	Remans et
AtUBQ10	GGCCTTGTATAATCCCTGATGAATAAG	AAAGAGATAACAGGAACGGAAACATAGT	al., 2008
AtHSP70	CCTACCAACACCGTCTTCGA	CTTCTCACCTGGACCGGAAA	
AtHSP18.2	CCGTTCTCGCAAGACTTATGG	GAAGCGTTTGCCAACGCAGAA	Ikeda et al.
AtHSFA2	GGAAGCAGCGTTGGATGTGA	TAGATCTTGGCTGTCCCAATCCA	2011
AtHSP21	ATGGACGTCTCTCCTTTCGGATT	CTGTTTCTTCCTGAGACAGGCATA	
AtLFG4	GCGTTTTTTTCCCCATTCGTC	GGGCTCTCAAGCATAGTCGGATA	
AtF22G5.11	TACGACGACGGAGATACGGTGAC	CCGCTTGGGAGGCTGCT	
AT1G16022	TCTCATTGGACTCCCAGAAACTCG	TTTTCCGCCTGTAACTCCACGAC	
AtF2E2.18	TCATCTTCTTCTTCCTCCGCC	ACAACATCGTTCCCTTCGTCAG	
AtBGLU34	CCAAAGAACCCTTCCACTGC	CCCCCAGTCAGTCTTCCCTT	
AtGOLS2	ACAAAGAAGATGGCACCTGAGA	CTTAGCCCCTTAGCAAGACCC	Designed
AtCSD2	TCGTCTTCTCATTCCTCCTTCC	TGAGTCATCTTGGGTCAAAGTAACA	
AtMYB102	AAGTGGTCTGCGATTGCGGC	TGGATGTTGCTGGTGATGACGAC	
AtFLP1	CACACACTCTACACGGTTTCCTTAT	CCTCATCCTCTGCTGCTGGTT	
AtT31P16.120	GGTGACGGGACCTGATGGG	CCTCTTGGCAGCGGGACA	
