# Supplementary material for: Heterologous VvDREB2c Expression Improves Heat Tolerance in Arabidopsis by Inducing Photoprotective Responses
Source: Int J Mol Sci. 2023 Mar 22;24(6):5989. doi: 10.3390/ijms24065989 (PMC10053783; doi:10.3390/ijms24065989)
Supplement: Supplementary file 1 [file ijms-24-05989-s001.zip › Table S2.pdf]

Table S2. Information on hormone standard samples

Name	Hormone	Molecular formula	Molecular weight
(+/-)-Abscisic acid	ABA	C15H20O4	264.32
1-AminocyclopropanecarbOEylic acid	ACC	C4H7NO2	101.105
3-aIndolecetic acid	IAA	C10H9O2	175.19
(+/-)-Jasmonic acid	JA	C12H18O3	210.27
Salicylic acid	SA	C7H6O3	138.12
