# Supplementary material for: Heterologous VvDREB2c Expression Improves Heat Tolerance in Arabidopsis by Inducing Photoprotective Responses
Source: Int J Mol Sci. 2023 Mar 22;24(6):5989. doi: 10.3390/ijms24065989 (PMC10053783; doi:10.3390/ijms24065989)
Supplement: Supplementary file 1 [file ijms-24-05989-s001.zip › Table S3.pdf]

Table S3. Linear equation table

Compound	Equation	R-squared
ABA	y=8.27e^4x-1.39e^4	0.9999
ACC	y=6.07e^4x+6.55^4	0.9999
IAA	y=5.26e^4x+7.57e^3	0.9994
JA	Y=4.8e^4x-1.76e^4	0.9998
SA	y=4.35e^5x+3.11e^5	1
