# Supplementary material for: Heterologous VvDREB2c Expression Improves Heat Tolerance in Arabidopsis by Inducing Photoprotective Responses
Source: Int J Mol Sci. 2023 Mar 22;24(6):5989. doi: 10.3390/ijms24065989 (PMC10053783; doi:10.3390/ijms24065989)
Supplement: Supplementary file 1 [file ijms-24-05989-s001.zip › Table S6.pdf]

Table S6. Number of genes in each module

Modules	Counts
turquoise	1320
green	220
blue	185
grey	110
brown	97
magenta	93
red	56
purple	27
