# Supplementary figures and images for: Heterologous VvDREB2c Expression Improves Heat Tolerance in Arabidopsis by Inducing Photoprotective Responses
Source: Int J Mol Sci. 2023 Mar 22;24(6):5989. doi: 10.3390/ijms24065989 (PMC10053783; doi:10.3390/ijms24065989)

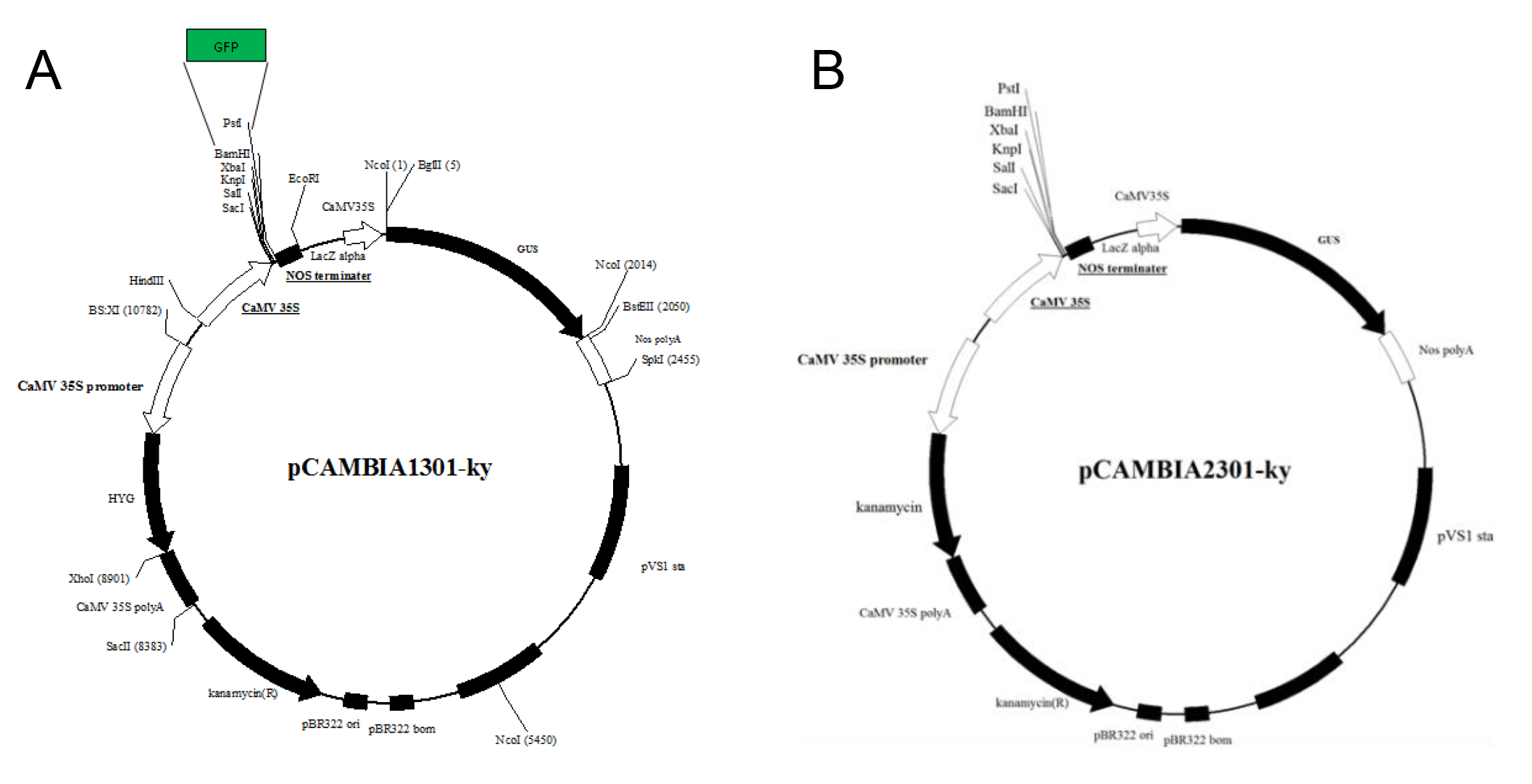

Supplement: Supplementary file 1 [file ijms-24-05989-s001.zip › Figure S1.tif]

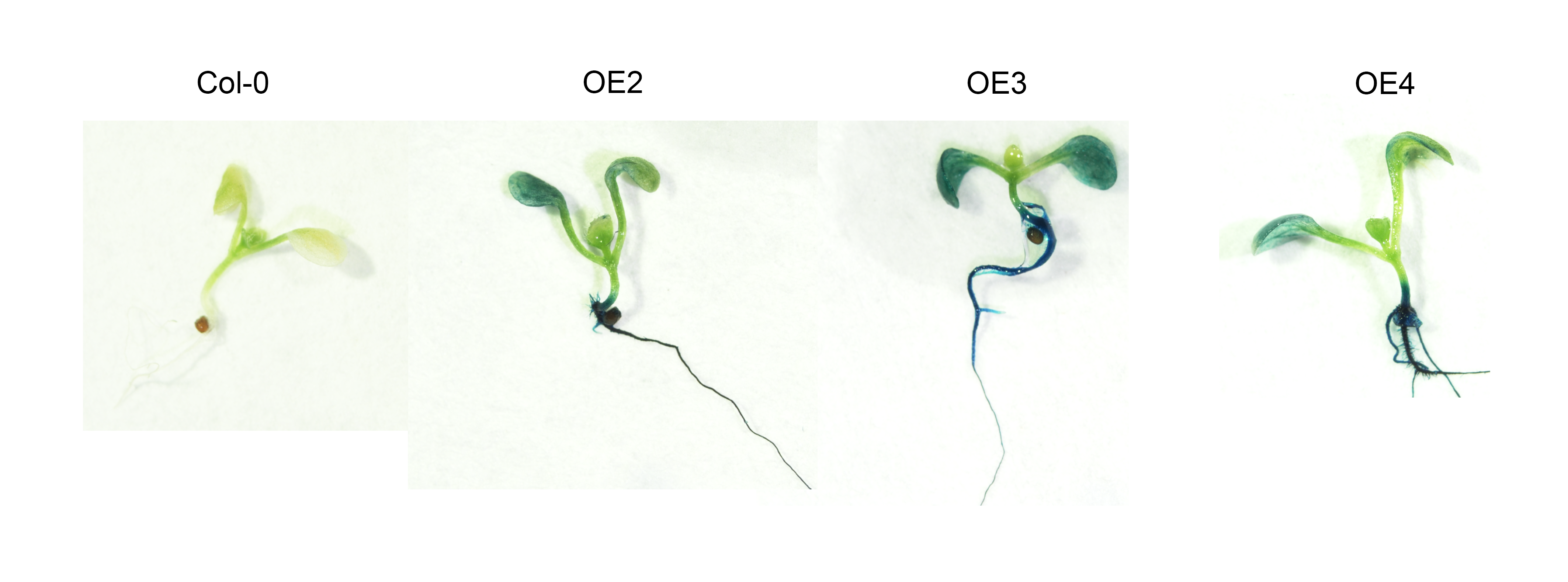

Supplement: Supplementary file 1 [file ijms-24-05989-s001.zip › Figure S2.tif]

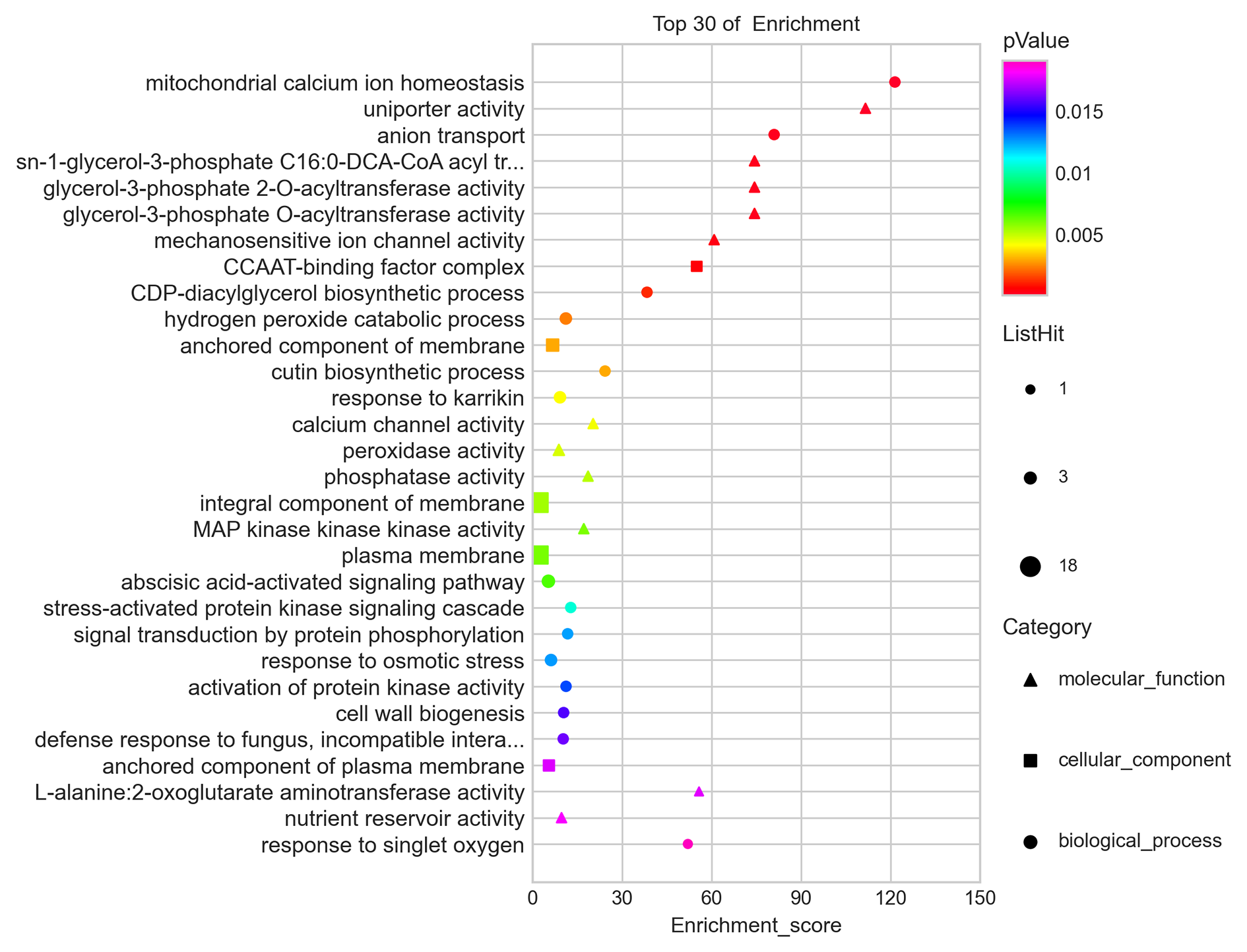

Supplement: Supplementary file 1 [file ijms-24-05989-s001.zip › Figure S3.tif]

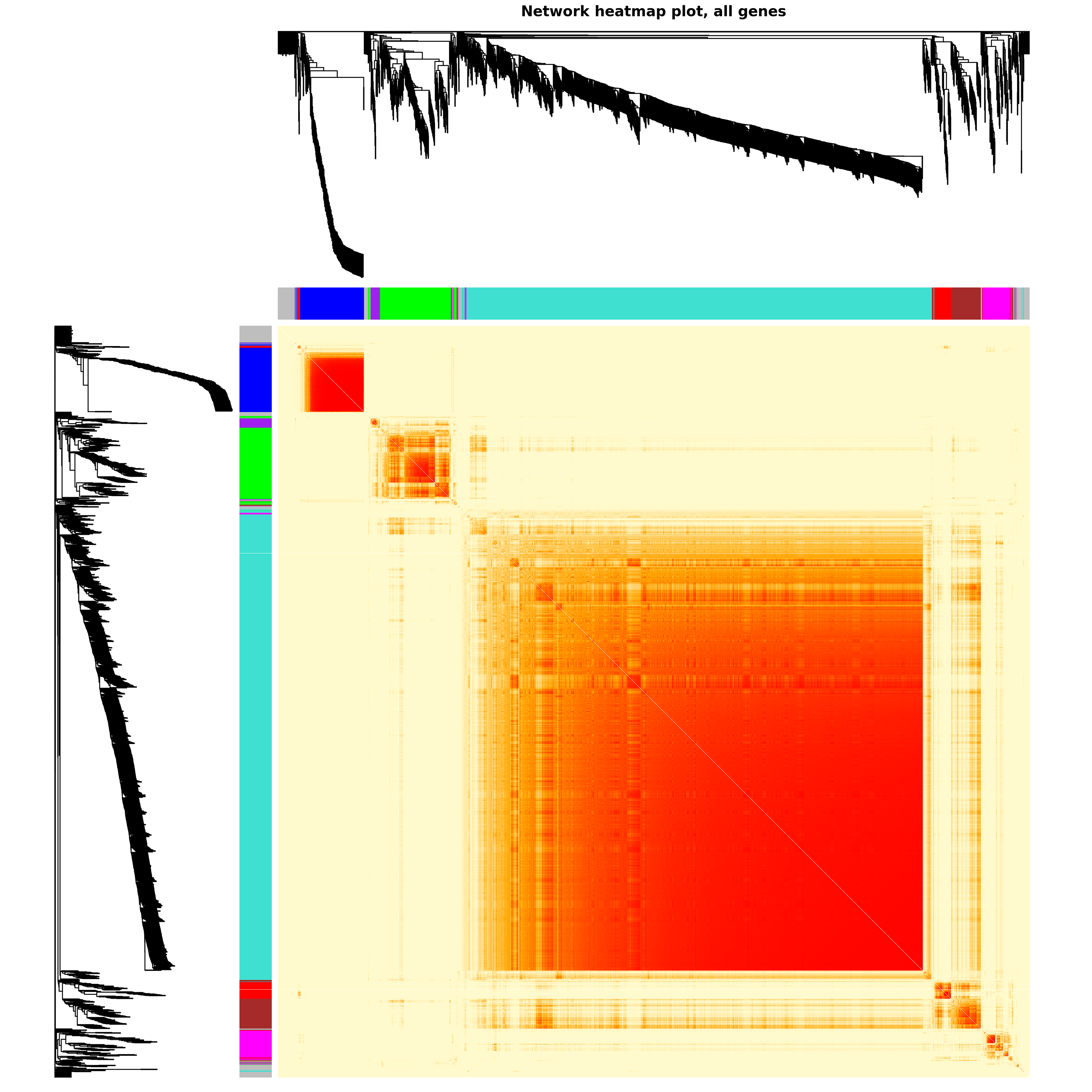

Supplement: Supplementary file 1 [file ijms-24-05989-s001.zip › Figure S4.tif]
